# TCEA1 Suppresses Acute Promyelocytic Leukemia by Upregulating C/EBPε and IRF8

**DOI:** 10.3390/ijms27125380

**Published:** 2026-06-15

**Authors:** Taomei Yang, Yonghu Wan, Chunwei Chu, Xiangyun Chen

**Affiliations:** 1 School of Basic Medicine, Guizhou University of Traditional Chinese Medicine, Guiyang 550025, China; yangtaomei017@gzy.edu.cn (T.Y.);; 2 Guizhou Center for Disease Control and Prevention, Guiyang 550004, China

**Keywords:** *TCEA1*, acute promyelocytic leukemia, *C/EBPε*, *IRF8*, granulopoiesis, animal model

## Abstract

We previously showed that *TCEA1* deficiency in myeloid cells promotes proliferation, impairs differentiation and inhibits apoptosis, but its role and underlying mechanism in acute myeloid leukemia (AML) are unknown. Here, in NB-4 cells, an M3 subtype of AML, *TCEA1* overexpression suppressed proliferation (*p* < 0.001), induced S-phase arrest (from 35.35% to 19.47%, *p* < 0.001), increased apoptosis (from 10.37% to 23.5%, *p* < 0.001), and promoted differentiation. Mechanistically, *TCEA1* overexpression upregulated *C/EBPε* and *IRF8* at the mRNA and protein levels; conversely, *TCEA1* knockdown downregulated both. Rescue experiments in *TCEA1* knockdown 32Dcl3 cells showed that ectopic *C/EBPε* or *IRF8* reversed the uncontrolled proliferation, blocked apoptosis, and impaired differentiation. In xenograft mouse models, *TCEA1* overexpression reduced leukemic infiltration in the bone marrow, spleen, and liver; extended overall survival; and elevated *C/EBPε* and *IRF8* expression in vivo. Analysis of public APL datasets revealed that high *TCEA1* expression is associated with a favorable prognosis (HR = 0.43, 95% CI: 0.2–0.93, logrank *p* = 0.028). Collectively, our findings demonstrate that *TCEA1* suppresses proliferation, promotes apoptosis and differentiation, and attenuates disease progression by upregulating *C/EBPε* and *IRF8*, positioning this regulatory mechanism as a potential therapeutic target and prognostic biomarker for this disease.

## 1. Introduction

Acute myelogenous leukemia (AML) is an aggressive hematologic malignancy characterized by the accumulation of immature myeloid blasts [1,2], arising from coordinated disruptions in differentiation, apoptosis, and proliferation [3,4,5]. Acute promyelocytic leukemia (APL) is a distinct subtype of acute myeloid leukemia (AML) defined by the t(15;17) translocation and the PML–RARA fusion oncoprotein, which blocks myeloid differentiation at the promyelocytic stage and confers apoptosis resistance, thereby driving leukemogenesis. The introduction of all-trans retinoic acid (ATRA) and arsenic trioxide (ATO) has transformed APL into a curable leukemia, yet early mortality and drug resistance remain significant challenges, highlighting a limitation of current therapies and underscoring the need for novel therapeutic targets and a deeper understanding of leukemogenesis.

Transcriptional dysregulation is a prominent feature observed in APL [6,7,8,9,10], with growing evidence implicating aberrant transcription elongation in the misregulation of key hematopoietic transcription factors that control myeloid development [11,12,13,14,15]. The transcription elongation factor TCEA1 (TFIIS) facilitates RNA polymerase II progression through arrest sites, thereby ensuring productive transcription elongation [16,17,18,19]. Our previous functional screen identified *TCEA1* as a critical regulator of myelopoiesis, with its knockdown in myeloid precursors leading to uncontrolled proliferation, blocked differentiation, and impaired apoptosis [20], hallmarks of hematopoietic malignancies. Bioinformatic analysis further revealed decreased *TCEA1* expression in lymphoma samples [20], raising the possibility that its dysfunction may extend to broader hematopoietic malignancies. CCAAT/enhancer-binding protein epsilon (*C/EBPε*) and interferon regulatory factor 8 (*IRF8*) are two pivotal transcription factors governing myeloid differentiation. *C/EBPε* is essential for terminal granulocytic differentiation, cell cycle arrest, and functional maturation [21,22,23]. *C/EBPε* regulates the transition from the promyelocytic stage to the myelocytic stage of neutrophil development and is indispensable for secondary and tertiary granule formation [24,25,26], as its deficiency leads to a block in neutrophil maturation. In APL, *PML–RARA* inhibits *C/EBPε* expression, whereas ATRA induces *C/EBPε* expression [8,27]. *IRF8* acts as a negative regulator of proliferation and is frequently downregulated in AML; its loss results in uncontrolled proliferation and predisposes to leukemic transformation [7,28,29,30]. In APL, *IRF8* acts as a tumor suppressor, and its downregulation by *PML–RARA* accelerates disease progression [7]. Notably, although ATRA and ATO are effective in APL, they fail to derepress *IRF8*, leaving this key transcription factor persistently suppressed [31]. Interestingly, public datasets analysis suggested that *TCEA1* knockdown could alter the expression of these factors [20], but a direct link in leukemia was unexplored. Given these observations, we hypothesized that *TCEA1* suppresses leukemic progression by regulating the expression of *C/EBPε* and *IRF8*, thereby inhibiting proliferation and promoting differentiation and apoptosis.

To test this hypothesis, we established a stable *TCEA1* overexpressing NB4 cell line (NB4-*TCEA1*). In vitro, *TCEA1* overexpression significantly suppressed proliferation, induced apoptosis, and promoted granulocytic differentiation. Mechanistically, *TCEA1* overexpression upregulated the expression of *C/EBPε* and *IRF8*, whereas *TCEA1* knockdown in 32Dcl3 downregulated these transcription factors, suggesting that *TCEA1* expression is critical for maintaining *C/EBPε* and *IRF8* levels. Importantly, rescue experiments in *TCEA1* knockdown 32Dcl3 cells revealed that forced expression of either *C/EBPε* or *IRF8* not only restored their own expression but also reversed the hyperproliferative phenotype and rescued the differentiation and apoptosis defects caused by *TCEA1* loss. To further validate these findings in vivo, we established a mouse xenograft model using NB4-*TCEA1* cells. *TCEA1* overexpression prolonged mouse survival, attenuated body weight loss, and reduced leukemic infiltration in the peripheral blood, liver, and spleen. Consistent with the in vitro observations, elevated expression of *C/EBPε* and *IRF8* was detected in the peripheral blood and tissues of NB4-*TCEA1* xenograft mice. Additionally, analysis of public APL datasets revealed a significant association between high *TCEA1* expression and improved overall survival. Collectively, our results identify *TCEA1* as an upstream regulator of *C/EBPε* and *IRF8* that controls proliferation, apoptosis, and differentiation in APL, further demonstrating that *TCEA1* overexpression suppresses leukemic progression in vivo and correlates with favorable prognosis in APL patients.

## 2. Results

### 2.1. Generation of a TCEA1 Over-Expressing Stable Leukemia Cell Line

**Figure 1:**
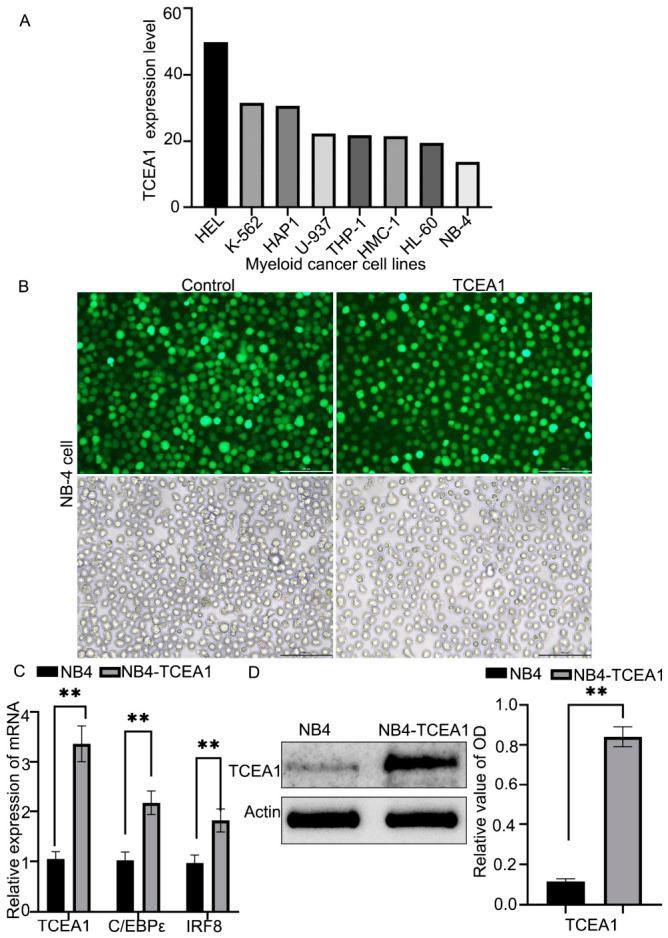
Establishment and validation of NB4 cell stably when overexpressing *TCEA1*. (**A**) *TCEA1* expression levels in various leukemia cell lines. Data were retrieved from the Human Protein Atlas database. Values represent single measurements from the database; error bars are not applicable. Cell lines: HEL (human erythroleukemia), K-562 (chronic myeloid leukemia, CML), HAP1 (near-haploid CML), U-937 (acute monoblastic/monocytic leukemia), THP-1 (acute monocytic leukemia), HMC-1 (mast cell leukemia), HL-60 (acute promyelocytic leukemia, APL), and NB-4 (acute promyelocytic leukemia, APL). Among the cell lines analyzed, NB4 cells exhibited relatively low basal expression of *TCEA1* compared with that of other leukemia cell lines examined. (**B**) Validation of *TCEA1* protein expression by fluorescence microscopy. Images show a uniform green fluorescent protein (GFP) signal in cells, indicating successful integration and stable expression of the recombinant construct. Scale bar = 100 μm. (**C**) qRT–PCR analysis of *TCEA1* mRNA expression. Total RNA was extracted from NB4 control and NB4-*TCEA1* cells, and *TCEA1* transcript levels were quantified by qPCR. Expression was normalized to housekeeping gene (*ACTB*). **, *p* < 0.01 based on Student’s *t*-test. (**D**) Western blot analysis of TCEA1 protein expression. Whole cell lysates were subjected to immunoblotting using an anti-TCEA1 antibody. The relative amounts of TCEA1 were quantified using ImageJ 1.54s software. The TCEA1 signals were normalized to the amount of β-actin. Data are presented as the means ± SD of three independent experiments. **, *p* < 0.01 based on the Student’s *t*-test.

To investigate the functional role of *TCEA1* in human leukemia, we first analyzed *TCEA1* expression levels across various leukemia cell lines using the Human Protein Atlas database. The results showed that NB4 cells exhibited a relatively low basal expression of *TCEA1* compared with that of other leukemia cell lines (Figure 1A). Using the NB4 cell line, we next generated a stable NB4 cell line overexpressing human *TCEA1*. Specifically, the full length of the *TCEA1* coding sequence was cloned and verified by sequencing. High-titer lentivirus was produced (1.5 × 10^8^ TU/mL for both *TCEA1* and control viruses). Following transduction and puromycin selection, a population exhibiting strong green fluorescence was visualized under fluorescence microscopy (Figure 1B). Overexpression of *TCEA1* was further confirmed at the molecular level by qPCR and at the protein level by Western blot analysis (Figure 1C,D). Collectively, these data confirm the successful generation of a stable NB4 leukemia cell line with a high level of *TCEA1* expression compared with that of NB4 control cells, providing a critical tool for subsequent functional studies.

### 2.2. TCEA1 Overexpression Inhibits Proliferative Capacity of Leukemia Cells

**Figure 2:**
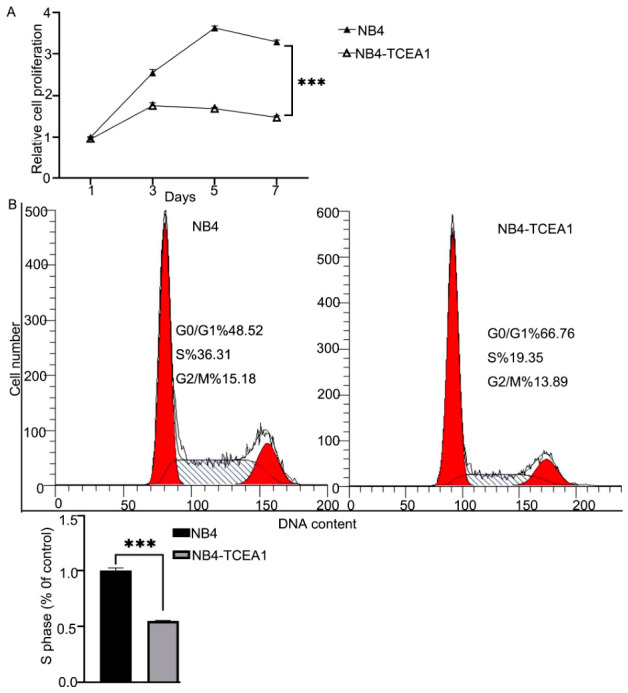
Effects of *TCEA1* overexpression on proliferation and cell cycle distribution in NB4 cells. (**A**) CCK-8 assay of cell proliferation. Cells were cultured for the indicated time points, and cell viability was assessed. Data are shown as mean ± SD from three independent experiments. Statistical significance was determined using Student’s *t*-test at each time point, ***, *p* < 0.001. (**B**) Cell cycle analysis by PI staining. Cells were cultured with NB4-specific medium, collected during logarithmic growth phase at 48 h post-seeding, stained with propidium iodide (PI), and then analyzed by flow cytometry to determine the percentage of cells in the G0/G1, S, and G2/M phases. Data from three independent experiments were normalized to the control and are presented as mean ± SD in the bar graph.. ***, *p* < 0.001 based on the Student’s *t*-test.

To assess the effect of *TCEA1* on leukemia cell proliferation, we performed CCK-8 assays. NB4 control and NB4-*TCEA1* cells were plated at equal densities and analyzed on days 1, 3, 5, and 7, using the same time points as in our loss-of-function experiments [20] to allow direct phenotypic cross-validation between the gain- and loss-of-function models. Overexpression of *TCEA1* significantly reduced the viability and proliferation rate of the NB4 cells compared with that of the control cells (Figure 2A). To determine whether this growth inhibition was associated with cell cycle alterations, we analyzed the cell cycle distribution via propidium iodide staining and flow cytometry. As shown in Figure 2B, NB4-*TCEA1* cells exhibited a marked decrease in the percentage of cells in the S phase (19.47% ± 0.11%) compared with that in the NB4 control cells (35.50% ± 0.88%), *p* < 0.001. These results, together with the proliferation data (Figure 2A), indicate that *TCEA1* functions as a negative regulator of cell proliferation in APL by impeding cell cycle progression.

### 2.3. TCEA1 Overexpression Promotes Apoptosis and Differentiation In Vitro

**Figure 3:**
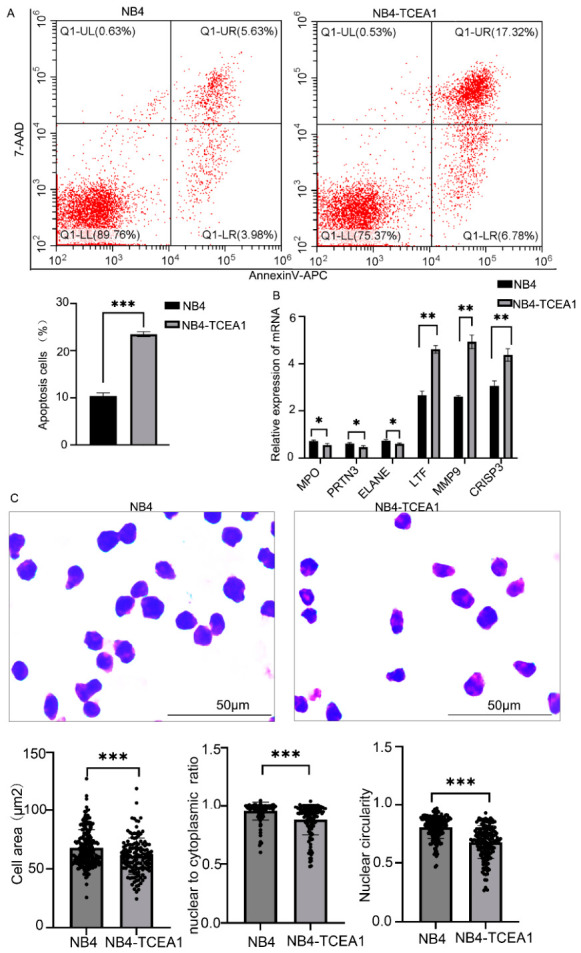
*TCEA1* overexpression promotes apoptosis and granulocytic differentiation in NB4 cells. (**A**) Apoptosis analysis by flow cytometry. NB4 control and NB4-*TCEA1* cells were cultured for 48 h in specific NB4 medium, then stained with Annexin V-APC and 7-AAD; apoptotic cells were quantified by flow cytometry. Representative images are shown, data from three independent experiments are presented as mean ± SD in the bar graph. ***, *p* < 0.001 based on the Student’s *t*-test. (**B**) Differentiation was assayed by qPCR. Total cellular RNAs were prepared and converted to first-strand cDNA. The relative expression of myeloperoxidase (*MPO*), neutrophil elastase (*ELANE*), proteinase 3 (*PRTN3*), lactoferrin (*LTF*), cysteine-rich secretory protein 3 (*CRISP3*), and gelatinase (*MMP9*) was normalized to ribosomal protein S16 RNA and analyzed by real-time PCR. Three independent experiments were analyzed by Graphpad Prism 11. Data are presented as mean ± SD. *, *p* < 0.05; **, *p* < 0.01, based on the Student *t*-test. (**C**) Morphological analysis. NB4 control and NB4-*TCEA1* cells were treated with 1 μM ATRA for 4 days and stained with Wright–Giemsa staining and examined by light microscopy. Representative images are shown (scale bar = 50 µm). For each group, at least 100 cells from three independent experiments were analyzed using ImageJ. Bar graphs show the cell area (µm^2^), nuclear-to-cytoplasmic (N/C) ratio, and cell circularity (mean ± SD). ***, *p* < 0.001 based on the Student’s *t*-test.

To further investigate the role of *TCEA1* in myeloid cell apoptosis, we assessed the apoptotic rate using Annexin V-APC/7-AAD double staining, followed by flow cytometric analysis. In addition to its inhibitory effect on proliferation, *TCEA1* overexpression significantly enhanced apoptosis in NB4 cells. Flow cytometry analysis revealed a marked increase in the percentage of Annexin V positive apoptotic cells in the NB4-*TCEA1* cells compared with that in the controls. Specifically, the apoptotic rate in the NB4 control cells was 10.37% ± 0.66%, whereas it increased to 23.50% ± 0.53% in the NB4-*TCEA1* cells, *p* < 0.001. Concurrently, the proportion of dead cells increased from 6.65% ± 0.40% to 16.31% ± 2.30%, *p* < 0.01 (Figure 3A). To determine whether *TCEA1* also influences leukemic differentiation, we assessed morphological changes and the expression of granule protein genes during all-trans retinoic acid (ATRA) treatment. Quantitative PCR analysis revealed that *TCEA1* overexpression altered the expression profile of granule protein genes. Compared with control cells, NB4-*TCEA1* cells showed a decreased expression of primary granule proteins myeloperoxidase (MPO), proteinase 3 (PRTN3), and neutrophil elastase (ELANE) and significantly increased expression of the secondary granule protein lactotransferrin (LTF), as well as the tertiary granule proteins matrix metalloproteinase 9 (MMP9) and cysteine-rich secretory protein 3 (CRISP3) granule protein transcripts (Figure 3B). All changes were statistically significant (*p* < 0.05, *p* < 0.01). Morphological analysis further demonstrated that control NB4 cells exhibited a typical primitive myeloid morphology characterized by large nuclei, a high nuclear-to-cytoplasmic ratio (0.95 ± 0.07), and round or oval shapes. In contrast, NB4-*TCEA1* cells exhibited differentiation features: reduced nuclear-to-cytoplasmic ratio (0.88 ± 0.13, *p* < 0.001), smaller area (60.75 ± 15.40 µm^2^), lower circularity (0.67 ± 0.14, *p* < 0.001), and nuclear segmentation (Figure 3C). These morphological findings, together with the molecular data (Figure 3B), confirm that *TCEA1* promotes APL cell differentiation. Collectively, these data indicate that *TCEA1* not only triggers apoptosis but also alleviates the differentiation block, thereby promoting both differentiation and apoptosis in this APL cell model.

### 2.4. TCEA1 Positively Regulates C/EBPε and IRF8 Expression

**Figure 4:**
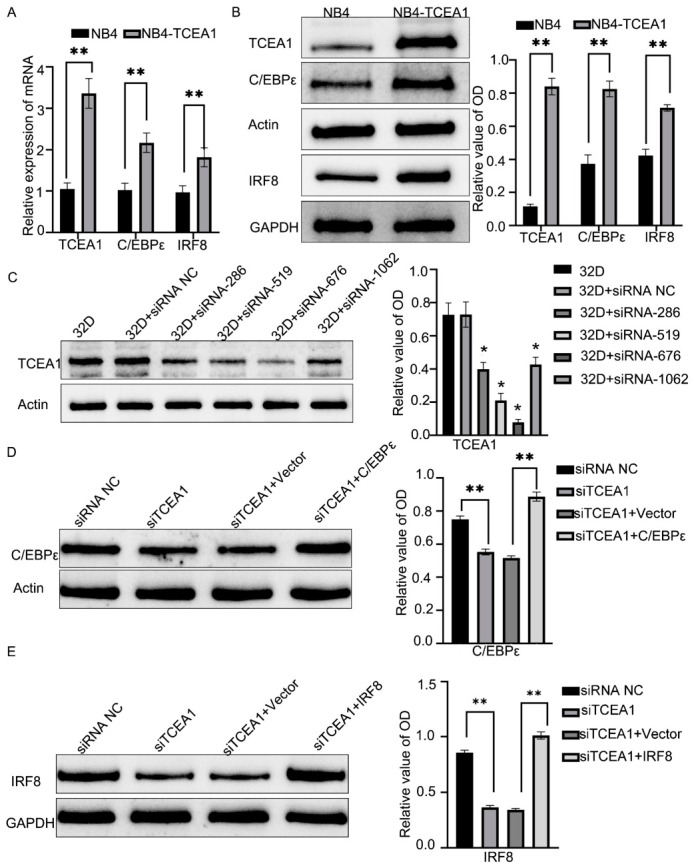
*TCEA1* positively regulates *C/EBPε* and *IRF8* expression. (**A**) qRT-PCR analysis of *C/EBPε* and *IRF8* mRNA levels in *TCEA1*-overexpressing NB4 cells. Cells were harvested, and total RNA was extracted. The mRNA levels of *C/EBPε* and *IRF8* were quantified by qPCR and normalized to *ACTB* and *GAPDH*, respectively. **, *p* < 0.01, Student’s *t*-test. (**B**) Western blot analysis of *C/EBPε* and *IRF8* protein levels in *TCEA1*-overexpressing NB4 cells. Whole-cell lysates were subjected to immunoblotting using antibodies against *C/EBPε* and *IRF8*, respectively. Protein bands were quantified using ImageJ software, and signals were normalized to β-actin and GAPDH, respectively. **, *p* < 0.01, based on the Student’s *t*-test. (**C**) Validation of *TCEA1* knockdown efficiency in 32Dcl3 cells. Cells were transfected with vectors expressing *TCEA1* siRNAs (si-RNA-286, 519, 676, 1062) or control siRNA. Total cellular lysates were subjected to Western blotting using an anti-TCEA1 antibody, with β-actin as a loading control. Relative TCEA1 protein levels were quantified using ImageJ software and normalized to β-actin, *, *p* < 0.05, based on the Student’s *t*-test. (**D**) *TCEA1* knockdown reduces *C/EBP*ε expression, and reintroduction of *C/EBP*ε rescues this effect. 32Dcl3 cells were transfected with control siRNA (lane 1) or siTCEA1 (lanes 2–4). *TCEA1*-knockdown cells were further transfected with an empty vector (lane 3) or a *C/EBPε* expression vector (lane 4). Protein levels of C/EBPε were assessed by Western blotting, with β-actin as a loading control. Band intensities were quantified using ImageJ software and normalized to β-actin. **, *p* < 0.01; Student’s *t*-test. (**E**) *TCEA1* knockdown reduces *IRF8* expression, and reintroduction of *IRF8* rescues this effect. IRF8 protein levels were assessed by Western blotting in the same set of cells described in (**D**), using an IRF8 specific antibody. Quantification and statistical analysis were performed as in (**D**). **, *p* < 0.01, based on the Student’s *t*-test.

Guided by bioinformatic analyses linking diminished *TCEA1* expression to reduced levels of the myeloid transcription factors *C/EBPε* and *IRF8* [20], we next examined whether *TCEA1* overexpression could conversely enhance their expression. Indeed, we found that enforced *TCEA1* expression in NB4 cells significantly upregulated both *C/EBPε* and *IRF8* mRNA levels (Figure 4A). Consistent with the transcriptional data, Western blot analysis confirmed a concomitant increase in C/EBPε and IRF8 protein abundance (Figure 4B). To further validate these results, we validated the knockdown efficiency of four *TCEA1* siRNAs in 32Dcl3 cells and selected the most effective siRNA-676, which was designated as siTCEA1 for subsequent experiments (Figure 4C). In 32Dcl3 cells with *TCEA1* knockdown, Western blot analysis demonstrated markedly reduced protein levels of *C/EBPε* (Figure 4D, lane 2) and *IRF8* (Figure 4E, lane 2) relative to those of the control cells (lane 1). To determine whether the reduction of *C/EBPε* and *IRF8* was specifically due to *TCEA1* loss, rescue experiments were performed by reintroducing *C/EBPε* or *IRF8* expression vectors into *TCEA1* knockdown cells. Notably, ectopic expression of *C/EBPε* (Figure 4D, lane 4) and *IRF8* (Figure 4E, lane 4) was successfully restored. These reciprocal findings indicate that *TCEA1* acts as a positive regulator of *C/EBP*ε and *IRF8*, establishing *TCEA1* as a key determinant of these critical granulocytic transcription factors.

### 2.5. TCEA1 Suppresses Proliferation and Promotes Apoptosis and Differentiation of Granulocytic Cells Through C/EBPε and IRF8

**Figure 5:**
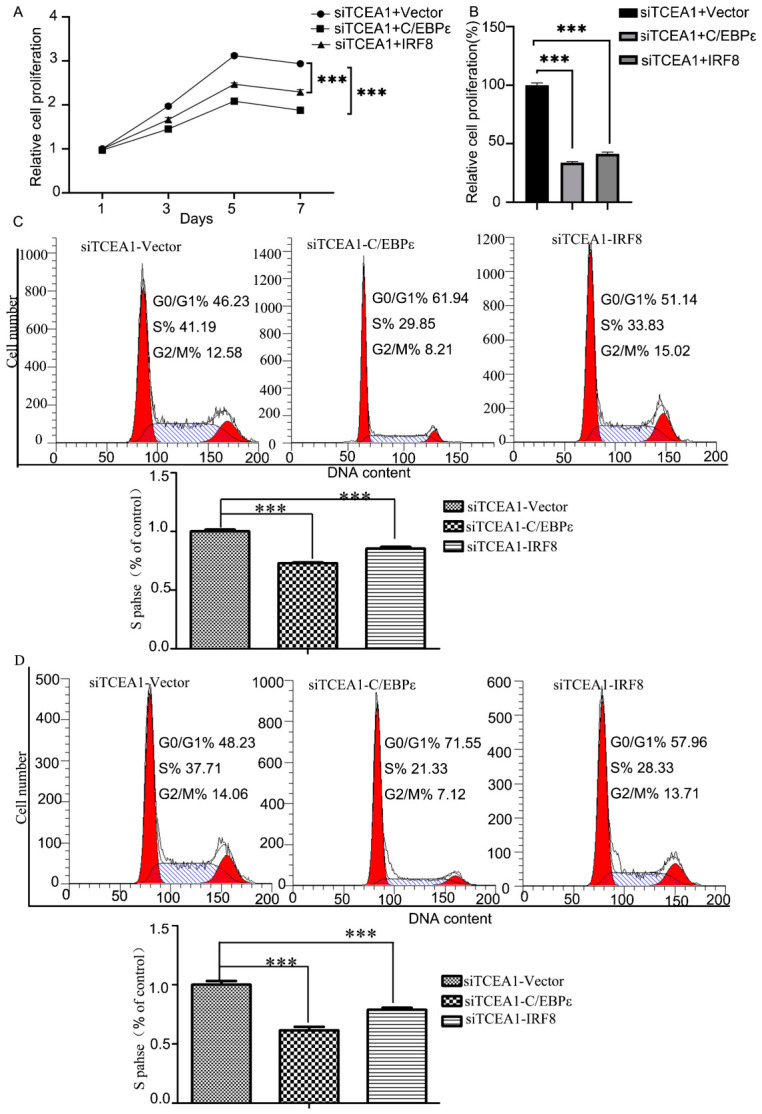
Re-expression of *C/EBPε* or *IRF8* rescues the uncontrolled proliferation and S-phase accumulation induced by *TCEA1* knockdown. (**A**) Rescue uncontrolled of proliferation under IL-3-containing conditions. 32Dcl3-si*TCEA1* cells were further transfected with *C/EBPε*- or *IRF8*-expressing vectors or empty vector controls. Cell viability was assessed by CCK-8 assay on days 1, 3, 5, 7 in medium containing IL-3. Data are shown as mean ± SD from three independent experiments. ***, *p* < 0.001 based on the Student’s *t*-test. (**B**) Rescue uncontrolled proliferation under G-CSF-induced differentiation conditions. Cells were cultured in medium containing G-CSF for 5 days, and cell viability was determined by CCK-8 assay. Data are shown as mean ± SD from three independent experiments. ***, *p* < 0.001 based on the Student’s *t*-test. (**C**) Cell cycle analysis under IL-3-containing conditions. Cells were cultured in IL-3-containing medium for 48 h, and cell cycle distribution was analyzed by flow cytometry. Representative images are shown. Data from three independent experiments were normalized to the control and are presented as mean ± SD in the bar graph. ***, *p* < 0.001 based on Student’s *t*-test. (**D**) Cell cycle analysis under G-CSF-induced differentiation conditions. Cells were cultured in G-CSF-containing medium for 48 h, and cell cycle distribution was analyzed. Representative images are shown. Data from three independent experiments were normalized to the control and are presented as mean ± SD in the bar graph.***, *p* < 0.001 based on Student’s *t*-test.

**Figure 6:**
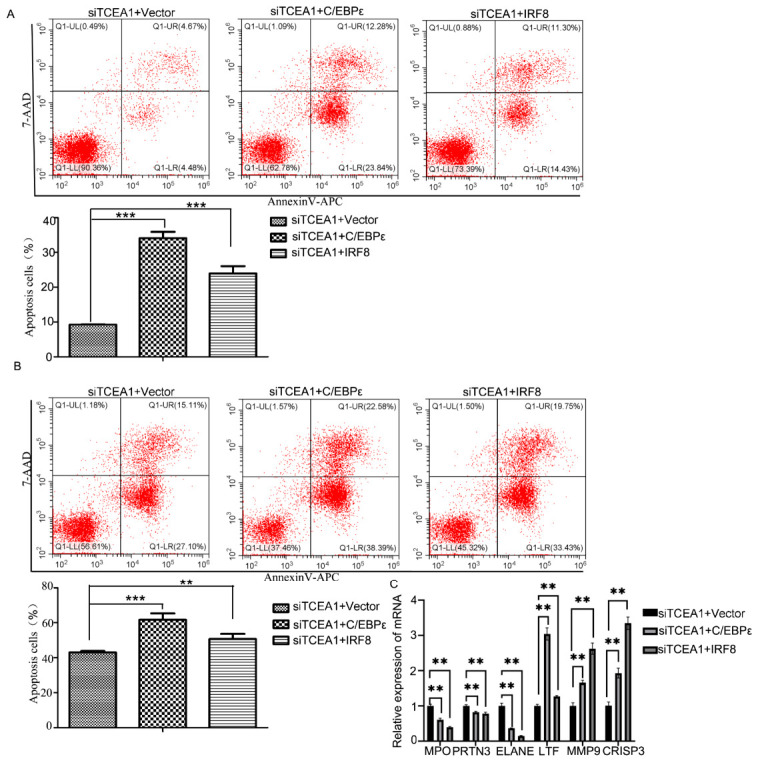
Re-expression of *C/EBPε* or *IRF8* rescues the apoptosis and granulocytic differentiation defects caused by *TCEA1* knockdown. (**A**) Apoptosis analysis under IL-3-containing conditions. 32Dcl3-si*TCEA1* cells expressing empty vector, *C/EBPε*, or *IRF8* were cultured in IL-3-containing medium for 48 h, and apoptosis was assessed by flow cytometry. Representative images are shown. Data from three independent experiments are presented as mean ± SD in the bar graph. ***, *p* < 0.001 based on Student’s *t*-test. (**B**) Apoptosis analysis under G-CSF-induced differentiation conditions. Cells were cultured in G-CSF-containing medium for 48 h, and apoptosis was determined by flow cytometry. Representative images are shown. Data from three independent experiments are presented as mean ± SD in the bar graph. **, *p* < 0.01. ***, *p* < 0.001 based on Student’s *t*-test. (**C**) Rescue of granulocytic differentiation. 32Dcl3-si*TCEA1* cells, with or without expression of *C/EBPε* or *IRF8*, were cultured for 5 days in medium containing G-CSF. Total cellular RNAs were prepared and converted to first-strand cDNA. The relative expression of myeloperoxidase (*MPO*), neutrophil elastase (*ELANE*), proteinase 3 (*PRTN3*), lactoferrin (*LTF*), cysteine-rich secretory protein 3 (*CRISP3*), and gelatinase (*MMP9*) was normalized to ribosomal protein S16 RNA and analyzed by real-time PCR. Data from three independent experiments are presented as mean ± SD. ** *p* < 0.01 based on the Student’s *t*-test.

To determine whether *C/EBPε* and *IRF8* mediate the functional effects of *TCEA1*, we performed rescue experiments in *TCEA1* knockdown 32Dcl3 cells by overexpressing *C/EBPε* or *IRF8*, with the corresponding empty vector as a control. The expression of *C/EBPε* or *IRF8* was confirmed prior to preforming the functional assays (Figure 4D,E). As shown, rescue expression of either *C/EBPε* or *IRF8* significantly suppressed the hyperproliferative phenotype induced by *TCEA1* knockdown when cells were cultured in interleukin-3 (IL-3) containing medium, as assessed by CCK-8 at days 1, 3, 5, and 7, *p* < 0.001 (Figure 5A). Similarly, under granulocyte colony-stimulating factor (G-CSF)-induced differentiation conditions, the cell viability significantly downregulated upon expression of either *C/EBPε* or *IRF8*, *p* < 0.001 (Figure 5B). Cell cycle analysis further revealed that, compared with the siTCEA1 + Vector control, re-expression of either *C/EBPε* or *IRF8* significantly reduced the proportion of cells in the S phase, under both IL-3 and G-CSF conditions (*p* < 0.001; Figure 5C and 5D, respectively). Apoptosis analysis (Figure 6A,B) showed that forced expression of *C/EBPε* or *IRF8* in *TCEA1* knockdown cells markedly increased the percentage of apoptotic cells compared with that of the vector control under both culture conditions (*p* < 0.01, *p* < 0.001). We next examined whether re-expression of *C/EBPε* or *IRF8* could rescue the differentiation arrest induced by *TCEA1* loss, as previously characterized [20]. Results (Figure 6C) showed that forced expression of either *C/EBPε* or *IRF8* in *TCEA1* knockdown cells significantly downregulated early granule proteins (MPO, PRTN3, ELANE) and upregulated secondary (LTF) and tertiary (MMP9, CRISP3) granule proteins (*p* < 0.01). These results demonstrate that *C/EBPε* and *IRF8* act downstream of *TCEA1* to promote granulocytic differentiation and apoptosis while inhibiting proliferation.

### 2.6. TCEA1 Suppresses Leukemia Progression In Vivo

To evaluate the in vivo role of *TCEA1* in myeloid leukemia progression, we established a xenograft mouse model. Recipient mice received a sublethal dose of total-body irradiation prior to cell injection to transiently suppress their residual innate immune activity and provide a permissive engraftment window for the injected cells. Subsequently, NB4 control cells or NB4-*TCEA1* cells were intravenously injected into the irradiated mice to establish an APL xenograft model.

#### 2.6.1. Survival Analysis

**Figure 7:**
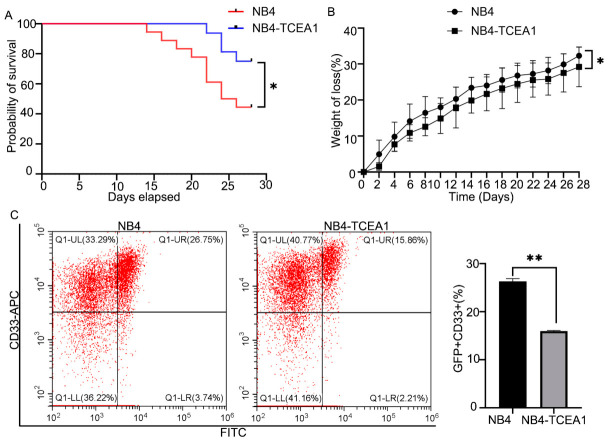
*TCEA1* overexpression improves survival and reduces leukemia burden in peripheral blood in a mouse xenograft model of APL. (**A**) Kaplan-Meier survival curves from three pooled independent experiments. NOD/SCID mice were injected with NB4 control or NB4–*TCEA1* cells in three independent experiments (*n* = 6 per group). The survival data from all three experiments were combined, yielding a total of 18 mice per group. Overall survival was monitored for 28 days. The median survival time was 25 days in the control group, and the median survival was not reached within the observation period of 28 days in the NB4-*TCEA1* group, *, *p* < 0.05 based on the log-rank test. (**B**) Body weight changes. Body weights of mice were measured at the indicated time points, and the percentage of weight loss relative to baseline was calculated. *, *p* < 0.05 based on the Student’s *t*-test. (**C**) Flow cytometric analysis of peripheral blood leukemia cell burden. Peripheral blood was collected from NB4 control or NB4-*TCEA1* mice at day 21 post-inoculation, and the percentage of GFP^+^CD33^+^ leukemic cells was determined by flow cytometry. **, *p* < 0.01 based on the Student’s *t*-test.

Kaplan–Meier survival analysis of pooled data from three independent experiments (total *n* = 18 per group) revealed that mice bearing NB4-*TCEA1* cells exhibited a significantly prolonged overall survival, with the median survival being 25 days in the control group, while it was not reached within the observation period of 28 days in the NB4-*TCEA1* group (Figure 7A, log-rank test, *p* = 0.04). Consistent with this, the percentage of body weight loss was significantly reduced in the *TCEA1* overexpressing group compared to the controls (Figure 7B, *p* < 0.05).

#### 2.6.2. Leukemia Cell Burden in Peripheral Blood

At day 21 post-inoculation, flow cytometric analysis was performed to assess the proportion of GFP^+^CD33^+^ leukemic cells in the peripheral blood. The *TCEA1* overexpressing group showed a significantly lower proportion (15.9% ± 0.2%) compared to that of the control group (26.3% ± 0.6%; **, *p* < 0.01; Figure 7C). Collectively, these results indicate that *TCEA1* overexpression reduces leukemic burden in peripheral blood.

#### 2.6.3. Inhibition of Organ Infiltration by TCEA1

**Figure 8:**
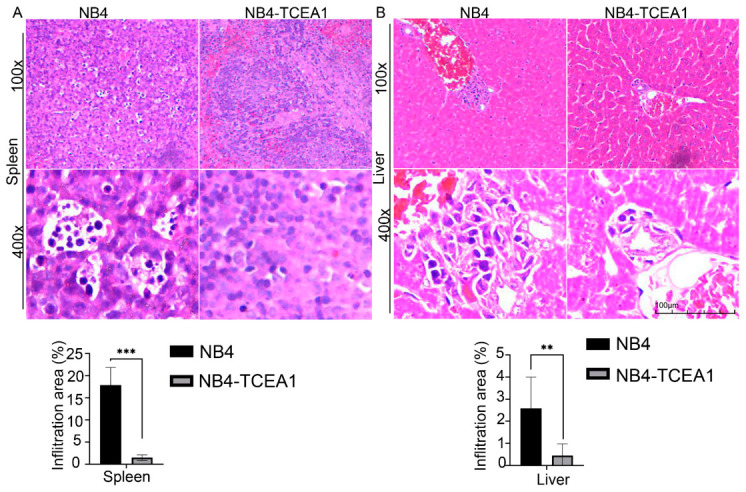
*TCEA1* overexpression suppresses extramedullary infiltration in the liver and spleen. (**A**) Histopathological analysis of the spleen. Hematoxylin and eosin (HE) staining was performed on spleens from mice bearing NB4 control or NB4-*TCEA1* xenografts. The area of leukemic infiltration was quantified using ImageJ software and is presented as mean ± SD in the bar graph (below). (**B**) Histopathological analysis of the liver. Hematoxylin and eosin (HE) staining was performed on livers from mice bearing NB4 control or NB4-*TCEA1* xenografts. The area of leukemic infiltration was quantified using ImageJ software and is presented as mean ± SD in the bar graph (below). Scale bars = 100 μm. **, *p* < 0.01; ***, *p* < 0.001 based on the Student’s *t*-test.

Histopathological analysis was performed to assess extramedullary infiltration. Macroscopically, neither group exhibited significant hepatosplenomegaly. Hematoxylin and eosin (HE) staining of liver and spleen tissues was performed to assess the impact of *TCEA1* on leukemic cell infiltration. In the spleen, tissue from control mice showed extensive architectural disruption by dense leukemic infiltrates. In contrast, spleens from the *TCEA1* overexpressing group displayed preserved structure, with markedly fewer infiltrating cells (Figure 8A). A parallel effect was observed in the liver. Control mice exhibited obvious leukemic cell infiltrates, which were substantially diminished in the *TCEA1* overexpressing group (Figure 8B). Collectively, these findings demonstrate that *TCEA1* effectively inhibits the tissue infiltration and dissemination of leukemia cells to major organs. This suppression of extramedullary infiltration complements its previously demonstrated effects on reducing peripheral blood tumor burden, underscoring its multifaceted role in attenuating leukemia progression in vivo.

#### 2.6.4. TCEA1 Upregulates C/EBPε and IRF8 Expression In Vivo

**Figure 9:**
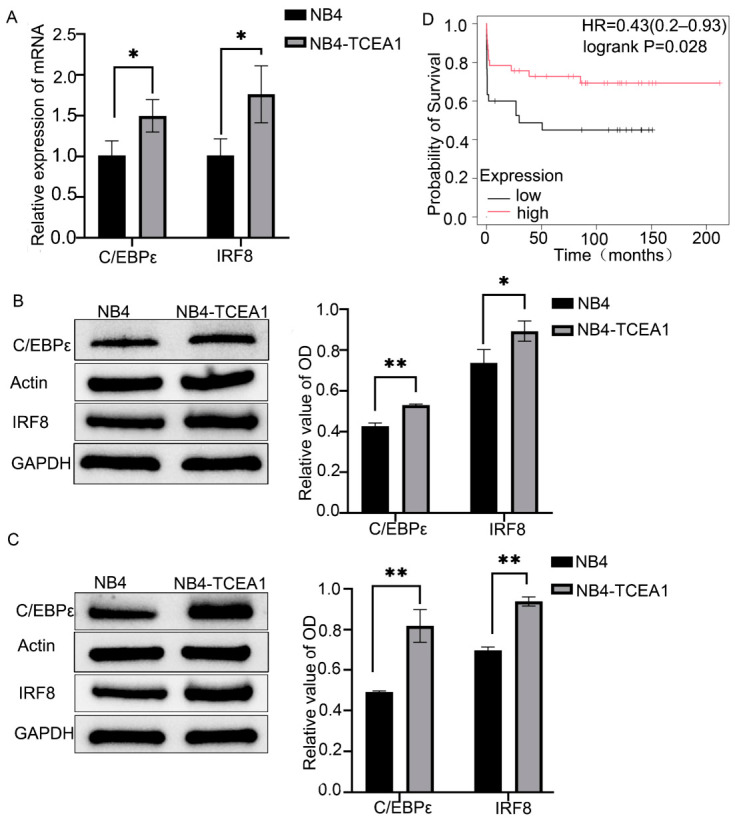
*TCEA1* upregulates *C/EBPε* and *IRF8* expression in vivo and correlates with favorable prognosis in APL patients. (**A**) qRT-PCR analysis of *C/EBP*ε and *IRF8* mRNA levels in peripheral blood leukocytes. RNA was isolated from peripheral blood leukocytes of xenograft mice. *C/EBPε* and *IRF8* mRNA levels were quantified by qPCR. *, *p* < 0.05 based on the Student *t*-test. (**B**,**C**) Western blot analysis of *C/EBP*ε and *IRF8* protein levels. Protein lysates were prepared from liver (**B**) and spleen (**C**) tissues of mice. Protein levels of C/EBPε and *IRF8* were assessed by immunoblotting, with β-actin and GAPDH as a loading control, respectively. *, *p* < 0.05, **, *p* < 0.01 based on the Student *t*-test. (**D**) Kaplan–Meier survival analysis of APL patients. Overall survival of APL patients (FAB M3 subtype) using the Kaplan–Meier Plotter online tool. Patients were stratified into low and high *TCEA1* expression groups based on the optimal cutoff value of 3975, which was determined by the “auto select best cutoff” algorithm that minimizes the log-rank *p* value within the interquartile range of the expression data. High *TCEA1* expression was associated with significantly better overall survival (HR = 0.43, 95% CI: 0.2–0.93, logrank *p* = 0.028). The upper quartile survival time was 38.7 months in the high-expression group compared with 0.5 months in the low-expression group.

Having established that *TCEA1* overexpression upregulates the key myeloid transcription factors *C/EBPε* and *IRF8* in vitro (Figure 4), we next investigated whether this program is engaged in vivo and examined its relevance to leukemic dissemination. To elucidate the molecular mechanism underlying *TCEA1* function in vivo, we examined *C/EBPε* and *IRF8* expression levels in tissues from xenograft mice. Quantitative PCR analysis of peripheral blood leukocytes revealed significantly elevated mRNA levels of both transcription factors in the *TCEA1* overexpressing group compared to that in the controls (Figure 9A). Consistently, Western blot analysis confirmed increased protein levels of *C/EBPε* and *IRF8* in both liver and spleen tissues from NB4-*TCEA1* mice (Figure 9B,C). These in vivo findings are consistent with our in vitro mechanistic studies, further validating that *TCEA1* exerts its biological functions by upregulating *C/EBPε* and *IRF8* expression.

### 2.7. High TCEA1 Expression Correlates with Favorable Prognosis in APL Patients

To further explore the clinical relevance of our findings, we next examined the correlation between *TCEA1* expression and patient outcomes using the Kaplan–Meier Plotter online tool. A total of 67 APL patients (FAB M3 subtype) were included in the analysis. Based on the optimal cutoff value of 3975, 37 patients were classified into the high-TCEA1 expression group and 30 into the low-expression group. High-*TCEA1* expression was associated with a lower risk of death (HR = 0.43, 95% CI: 0.2–0.93, logrank *p* = 0.028, Figure 9D). Kaplan–Meier analysis revealed that the upper quartile survival time was 38.7 months in the high-expression group compared with only 0.5 months in the low-expression group. These findings align with our experimental data showing that *TCEA1* suppresses proliferation and promotes differentiation in APL cells.

## 3. Discussion

This study establishes *TCEA1* as a critical regulator that supports proper myeloid differentiation, induces apoptosis, and inhibits leukemic proliferation in human APL. Using the NB4 cell line, which we found to exhibit low basal *TCEA1* expression and which represents the M3 subtype of AML, we demonstrate that elevating *TCEA1* levels suppresses the malignant phenotype both in vitro and in vivo. In particular, *TCEA1* overexpression inhibits proliferation, induces differentiation, and promotes apoptosis. Differentiation blockade in APL is associated with dysregulated expression of key myeloid transcription factors, including *C/EBPε* and *IRF8*, which are essential for granulocytic maturation [9,27,31]. Thereby, our work positions *TCEA1* as a guardian of the transcriptional programs required for myeloid maturation. Mechanistically, this function is mediated through its positive regulation of the transcription factors *C/EBPε* and *IRF8*. As is known, *C/EBPε* is a master regulator of terminal granulopoiesis, and its deficiency leads to myelodysplasia and blocks differentiation [21,27,32,33]. Consistent with this, *C/EBPε* expression is known to be suppressed in APL. ATRA, a differentiating agent to which APL is particularly sensitive, induces APL differentiation specifically through upregulating *C/EBPε* [27]. *IRF8* acts as a brake on proliferation, and its loss is associated with APL [7,28,34]. However, ATRA and ATO are unable to relieve *PML/RARα*-mediated repression of *IRF8*, leaving its expression refractory to current differentiation therapies [31]. Using complementary gain- and loss-of-function approaches, we showed that *TCEA1* positively regulates *C/EBPε* and *IRF8* expression, as evidenced by upregulation following *TCEA1* overexpression and downregulation following its knockdown. Crucially, rescue experiments, in which re-expression of *C/EBPε* or *IRF8* reversed the differentiation arrest, hyperproliferation, and apoptosis defect caused by *TCEA1* knockdown, confirm that these transcription factors act as critical downstream mediators of *TCEA1*′s function. Given that *IRF8* activates the pro-apoptotic gene *Bax* and represses the anti-apoptotic genes *Bcl-xL* and *Bcl-2* in myeloid cells [35,36,37], the increased apoptosis observed upon *TCEA1* overexpression is likely mediated through *IRF8*-dependent regulation of these *Bcl-2* family members. Thus, *TCEA1* exerts its pro-differentiation, anti-proliferative, and pro-apoptotic effects through positive regulation of *C/EBPε* and *IRF8*. Notably, unlike ATRA or ATO, *TCEA1* can effectively restore *IRF8* expression, which otherwise remains refractory to current therapies, highlighting *TCEA1* as a promising target for overcoming differentiation resistance in APL.

In interpreting the antiproliferative and pro-apoptotic effects described above, CCK-8 was monitored at the same time points (1, 3, 5, and 7 days) used in our loss-of-function experiments [20], which did not include a dedicated 48 h measurement. At 48 h, *TCEA1* overexpression increased the apoptotic rate and the proportion of dead cells (Figure 3A). Because a CCK-8 measurement was not taken at this time point, the reduced signal alone cannot directly distinguish whether it reflects genuine proliferation arrest or is secondary to the increased cell death. However, several lines of evidence indicate that both mechanisms contribute independently. The estimated net reduction in cell expansion at 48 h, derived from the CCK-8 curve, substantially exceeded the increase in either apoptotic or dead cells alone. Moreover, the S-phase fraction decreased markedly at the same time point (Figure 2B), providing an apoptosis-independent confirmation of impaired cell cycle progression. These observations argue that the loss of the CCK-8 signal reflects genuine proliferation arrest, in addition to cell death, and the sustained decrease over the experimental period is best understood as the cumulative outcome of concurrent antiproliferative and pro-apoptotic mechanisms.

Consistent with these observations, *TCEA1* overexpression in a xenograft model led to a marked reduction in disease burden. This was evidenced by diminished infiltration into the spleen and liver, accompanied by reduced leukemic burden in the peripheral blood. Such suppression of extramedullary dissemination is particularly notable, as it suggests a role for *TCEA1* in limiting the invasive potential of leukemic cells.

Emerging evidence has established that transcriptional elongation control plays a pivotal role in myeloid lineage commitment and leukemogenesis. In granulocytic differentiation, proper RNA polymerase II (RNAPII) pause–release dynamics are essential for the timely expression of lineage-specific transcription factors [38,39,40,41]. Indeed, RNAPII promoter-proximal pausing is a key regulatory event in transcription, and its dysregulation impairs the precise temporal expression of genes required for cell-fate decisions [40,41,42]. Lineage-specific transcription factors such as *C/EBPε* and *IRF8*, which govern granulopoiesis [24,33,43,44,45,46], are subjected to such regulatory control, as their expression must be precisely timed during myelopoiesis. Accordingly, their dysregulation, whether through aberrant transcriptional elongation or other mechanisms, is responsible for differentiation arrest and proliferation promotion. Notably, aberrant elongation control has been implicated in the pathogenesis of other myeloid malignancies, particularly in *MLL*-rearranged leukemias. An oncogenic principle underlying these leukemias is the pathological hijacking of the RNA polymerase II transcriptional elongation regulation [47,48]. MLL fusion proteins function as aberrant scaffolds that constitutively recruit the Super Elongation Complex (SEC) and its catalytic engine *CDK9* to oncogenic loci to drive the sustained expression of leukemogenic drivers such as *HOXA9* and *MEIS1* [47,48,49,50]. This transcriptional addiction state results in the sustained expression of key hematopoietic regulators, which in turn block differentiation, confer apoptosis resistance, and promote uncontrolled proliferation [50].

In contrast to this oncogenic mechanism in other myeloid malignancies, our findings reveal a distinct role for *TCEA1* in APL. Rather than driving leukemogenic gene expression, *TCEA1* facilitates the expression of myeloid differentiation-associated transcription factors *C/EBPε* and *IRF8*, thereby suppressing APL progression. These findings, together with the established role of *TCEA1* in transcriptional elongation [16,17], add a new insight into the understanding of transcriptional regulation in APL, revealing a functional role for *TCEA1* in promoting the expression of key transcription factors that govern myeloid differentiation and restrict leukemic progression. Notably, given that *IRF8* is refractory to ATRA/ATO [31], *TCEA1*-mediated upregulation of *IRF8* offers a potential therapeutic strategy for resistant APL. Together, these findings position *TCEA1* as a functionally unique regulator within the transcriptional elongation network, pointing to its potential as a therapeutic target.

Several limitations of this study should be considered. Our functional and mechanistic insights are primarily derived from a gain-of-function model in the NB4 cell line, which represents the M3 subtype of AML. While this provides a valuable proof-of-concept, further validation in a broader panel of APL or other AML cell lines representing non-M3 subtypes, as well as in primary patient-derived samples, is necessary to confirm the generalizability of our findings, and whether *TCEA1* plays a similar role in non-M3 AML subtypes remains an open question for future investigation. Additionally, in vivo survival analysis confirmed that *TCEA1* overexpression significantly prolonged overall survival, with the median survival not reached in the *TCEA1* group within the observation period, compared to a median survival of 25-day in the control group. Because the observation was terminated before the *TCEA1* group reached median survival, the true survival benefit may be even greater than that captured here. Therefore, studies with extended observation periods would be valuable to precisely quantify the full extent of survival prolongation and to assess long-term outcomes. With respect to the experimental design, although an irradiation-only control group was not included, sublethal irradiation (3 Gy) in NOD/SCID mice has been shown not to cause mortality over observation periods of 9 weeks and 20 weeks, respectively [51,52], both substantially exceeding the 28-day duration of our experiment. Given that sublethal irradiation alone is insufficient to cause mortality within this timeframe, the survival differences observed between the NB4-control and NB4-*TCEA1* groups reflect differential leukemia progression rather than irradiation-related toxicity. Furthermore, our in vitro loss-of-function studies were conducted in murine cells, which, while complementary, do not fully recapitulate the human disease context. Future studies using conditional knockout models will be essential to delineate the physiological role of *TCEA1* in normal hematopoiesis and its contribution to leukemogenesis in vivo. Mechanistically, although we have identified *C/EBPε* and *IRF8* as key downstream effectors, the further precise molecular mechanism by which *TCEA1* facilitates their transcriptional elongation requires further investigation. Advanced techniques such as precision nuclear run-on sequencing (PRO-seq) and chromatin immunoprecipitation (ChIP) will be critical to determine whether *TCEA1* directly binds to and promotes RNA polymerase II processivity at these target loci. Finally, while our bioinformatic analysis using publicly available datasets revealed a significant association between high *TCEA1* expression and improved overall survival in APL patients, this correlative finding requires validation in independent, prospectively collected clinical cohorts to fully ascertain its prognostic and translational potential.

In conclusion, our study establishes *TCEA1* as a critical regulator of myeloid proliferation, differentiation, and apoptosis in APL. We demonstrate that *TCEA1* functions by upregulating the expression of the key transcription factors *C/EBPε* and *IRF8*, thereby promoting granulocytic maturation and restraining leukemic phenotypes. Complemented by bioinformatic data linking *TCEA1* expression to favorable clinical outcomes, this work identifies *TCEA1* as an important modulator of myeloid homeostasis whose dysregulation contributes to APL pathogenesis, establishing it as a potential therapeutic target.

## 4. Materials and Methods

### 4.1. Cell Culture

The human acute promyelocytic leukemia cell line NB4 was purchased from Procell (cat. no. CL-0676, Wuhan, China) and was maintained in NB4-specific medium (cat. no. ZM0553, Zqxzbio, Shanghai, China), consisting of RPMI1640 supplemented with 10% fetal bovine serum (FBS) and 1% penicillin–streptomycin (100 U/mL penicillin, 100 μg/mL streptomycin). For differentiation studies, cells were treated with 1 µM all-trans retinoic acid (ATRA, cat. no. HY-14649, MedChemExpress, Monmouth Junction, NJ, USA). The human embryonic kidney 293 cells expressing the SV40 large T antigen (HEK293T) packaging cell line was cultured in Dulbecco’s Modified Eagle Medium (cat. no. 11965092, Thermo Fisher Scientific, Waltham, MA, USA) supplemented with 10% fetal bovine serum (cat. no. 16000044, Thermo Fisher Scientific). 32Dcl3, a murine myeloid progenitor cell line that proliferates in an IL-3-dependent manner and differentiates into granulocytes upon G-CSF stimulation, was cultured in growth medium containing IL-3 (2 ng/mL, cat. no. 213-13, PeproTech, Cranbury, NJ, USA) and differentiation medium containing G-CSF (50 ng/mL, cat. no. 250-05, PeproTech), as described previously [20]. All cell lines were incubated at 37 °C in a humidified atmosphere of 5% CO_2_.

### 4.2. Generation of TCEA1-Overexpressing Stable Cell Line

A lentiviral system was employed to generate NB4 cells stably overexpressing human *TCEA1*. The full-length human *TCEA1* coding sequence was cloned into the pHBLV-CMV-MCS-EF1-ZsGreen-T2A-puro lentiviral vector (cat. no. LV083, Hanbio Biotechnology, Shanghai, China). The recombinant plasmid, along with the packaging plasmids psPAX2 and pMD2.G, was co-transfected into HEK293T cells using Lipofiter™ transfection reagent (cat. no. 11668091, Thermo Fisher Scientific). Viral supernatant was harvested at 48 and 72 h post-transfection, concentrated by ultracentrifugation, and resuspended in a small volume of culture medium. Viral titer was determined on 293T cells and reached 1.5 × 10^8^ transducing units (TU)/mL. For transduction, NB4 cells were incubated with the concentrated *TCEA1*-overexpressing lentivirus or a control virus (carrying the empty vector) in the presence of polybrene (8 µg/mL, cat. No.TR1003, Sigma-Aldrich, St. Louis, MO, USA). At 24 h post-transduction, the medium was replaced with fresh complete medium. Stable polyclonal populations were selected with puromycin (1 µg/mL, cat. no. P4512, Sigma-Aldrich) for one week. Successful overexpression was confirmed by visualization of green fluorescence and quantified by qRT–PCR and Western blot analysis of *TCEA1* expression.

### 4.3. Cell Proliferation and Viability Assay

Cell proliferation and viability were assessed using the Cell Counting Kit-8 (cat. no. HY-K0301, MedChemExpress LLC, Monmouth Junction, NJ, USA). Cells were seeded in 96-well plates at a density of 5 × 10^3^ cells per well and cultured for 1, 3, 5, and 7 days. To avoid nutrient depletion and medium acidification during the prolonged 7-day culture, 50% of the culture medium was gently removed and replaced with an equal volume of fresh medium every other day. At the indicated time points, 10 µL of CCK-8 reagent was added to each well. After incubation at 37 °C for 2–4 h, the absorbance at 450 nm was measured using a microplate reader (MultiSkan FC, Thermo Fisher Scientific, Waltham, MA, USA). Experiments were performed in triplicate wells and repeated at least three times independently. Data were normalized to the control group at 24 h (set as 100%) to calculate relative proliferation rates.

### 4.4. Cell Cycle Analysis

Cells in the logarithmic growth phase after 48 h of culture were harvested, washed twice with phosphate-buffered saline (PBS), and fixed in 70% ice cold ethanol at 4 °C for 2 h. After fixation, cells were washed with PBS and then resuspended in the staining solution containing PBS, 200 µg/mL RNase A (cat. no. R8020, SOLARBIO, Beijing, China), and 50 µg/mL propidium iodide (PI; cat. no. P4170, Sigma-Aldrich). Cells were incubated in this staining solution at 37 °C for 20 min in the dark. DNA content was analyzed immediately using a flow cytometer (Beckman Coulter, Brea, CA, USA). Cell cycle phase distribution was quantified from the DNA content using Cell Quest software (MultiCycle for Windows 32-bit).

### 4.5. Cell Apoptosis Assay

Cell apoptosis was analyzed by flow cytometry using an annexin V-APC/7-AAD detection kit (cat. no. KGA1106-100, KeyGene Biotech, Nanjing, China), according to the manufacturer’s instructions. Cells cultured for 48 h were harvested, washed with PBS, and resuspended in binding buffer. Cells were stained with APC-conjugated annexin V and 7-AAD in the dark for 15 min at room temperature, diluted with additional buffer, and analyzed immediately by flow cytometry. Viable (annexin V-APC^−^/7-AAD^−^), early apoptotic (annexin V-AP^+^/7-AAD^−^), and late apoptotic (annexin V-APC^+^/7-AAD^+^) populations were quantified (Beckman coulter cytoFLEX). Experiments were performed in triplicate and repeated three times independently.

### 4.6. Morphologic Analysis In Vitro

NB4 and NB4-*TCEA1* cells were treated with 1 μM all-trans retinoic acid (ATRA, cat. no. HY-14649, MedChemExpress) or vehicle control (DMSO) for 4 days, changing the 40% medium every day. Cells were harvested, and blood smears were prepared. After air drying, slides were stained with Wright–Giemsa stain solution (cat. no. G1007, Servicebio, Wuhan, China), according to standard protocols. Specifically, slides were stained with Wright’s stain for 1 min, followed by Giemsa stain for 30 s, and then Wright–Giemsa C solution for 2 min, with gentle rinsing using distilled water between each step. Cellular morphology was evaluated by light microscopy (Nikon, Tokyo, Japan), and representative images were captured. Quantitative morphological analysis was performed using ImageJ: for each group, at least 100 cells from three independent experiments were manually delineated to measure cell area (μm^2^), cell circularity, and nuclear-to-cytoplasmic (N/C) ratio. Differentiation was assessed based on these parameters.

### 4.7. Quantitative Real-Time PCR

Total RNA was extracted using TRIzol reagent (cat.no. R401-01, VAZYME, Nanjing, China)). cDNA was synthesized from 1 µg of RNA using the HiScript II Q RT SuperMix (cat. no. R223-01, Vazyme). Quantitative PCR was performed in triplicate on a Real-Time PCR System (QuantStudio 6) with SYBR Green Master Mix (cat. no. R223-01, Vazyme). Primer sequences are summarized in Supplementary Table S1.

### 4.8. Western Immunoblots

Cells were cultured for 48 h, and protein lysates were extracted from exponentially growing cells using RIPA lysis buffer (cat. no. MA0151, Meilunbio, Dalian, China) supplemented with protease inhibitor cocktail (cat. no. MB12707, Meilunbio). Protein concentrations were determined using a BCA assay (cat. no. G3422, GBCBIO, Guangzhou, China). Equal amounts of protein (30 µg per lane) were separated by SDS-PAGE on 10% gels and transferred to PVDF membranes (cat. no. IPVH00010, Millipore, Burlington, MA, USA). Membranes were blocked with 5% BSA in TBST for 2 h at room temperature and subsequently incubated overnight at 4 °C with primary antibodies diluted in blocking buffer. The following primary antibodies were used: anti-TCEA1 (1:1000, cat. no. 17825-1-AP, Proteintech, Wuhan, China), anti-C/EBPε (1:1000, cat. no. PA5-36094, Thermo Fisher), and anti-IRF8 (1:1000, cat. no. 18977-1-AP, Proteintech,). After washing, membranes were incubated with an HRP-conjugated secondary antibody for 1 h at room temperature. Membranes were then incubated with HRP-conjugated secondary antibodies: goat anti-mouse IgG (H + L)-HRP (1:10,000, cat.no. BA1051, Wuhan Boster Biological Technology, Wuhan, China) and goat anti-rabbit IgG (H+L)-HRP (1:10,000, cat. no. BA1054, Wuhan Boster Biological Technology). Protein bands were visualized using an enhanced chemiluminescence (ECL) luminescent liquid (Super ECL Chemiluminescence Substrate Detection Kit, cat. no. PMK003, BioPM, Beijing, China). The membranes were imaged with a ChemiDoc MP imaging system (Model SH-523, Shenhua Technology, Hangzhou, China). Band intensities were quantified using ImageJ software and normalized to β-actin (1:5000, cat. no. 66009-1-Ig, Proteintech) or GAPDH (1:5000, cat. no. 66009-1-Ig, Proteintech).

### 4.9. NB4-TCEA1 Xenograft Model

All animal procedures were approved by the institutional animal care and use committee of Guizhou University of Traditional Chinese Medicine (approval no. 2025022). The mice (NOD/SCID, 6–8 weeks old, female) were purchased from SPF (Beijing) Biotechnology (Beijing, China). After 5 days of acclimatized, the mice were randomly assigned to two groups: the NB4 control group and the NB4-*TCEA1* group. Inclusion criteria for all mice were healthy appearance, normal activity, body weight within ±20%, and absence of any visible abnormalities. The experiment was performed three times independently, with each experiment containing six mice per group. (the NB4 control group and the NB4-*TCEA1* group). The mice received a sublethal dose of total body irradiation (3 Gy) [51,52] using Elekta Axesse. Within 24 h post-irradiation, the mice were injected via the tail vein with 1 × 10^7^ NB4-*TCEA1* or NB4 control cells in 0.2 mL PBS. Following transplantation, the mice were housed under specific pathogen-free conditions with autoclaved food, water, and bedding. The mice were monitored daily for weight, activity, and signs of illness. The mice were euthanized at defined endpoints or upon meeting humane endpoints. Peripheral blood, spleens, and livers were harvested.

### 4.10. Analysis of Leukemic Cell Infiltration in Peripheral Blood

Peripheral blood was collected from xenograft model mice at day 21 post-inoculation [53]. Red blood cells were lysed using ACK lysis buffer (cat. no. A1049201, Thermo Fisher Scientific). The remaining leukocytes were washed, counted, and resuspended in staining buffer (PBS containing 2% FBS). Cells were incubated with an anti-human CD33 antibody (cat. no. 17425-1-AP, Proteintech) at a predetermined optimal dilution for 30 min at 4 °C in the dark, followed by washing. Cell populations were analyzed on a flow cytometer (cytoFLEX, Beckman coulter). Leukemic cells were identified as GFP^+^ (from the transduced NB4 cells) and CD33^+^ (human myeloid marker) double-positive events. The percentage of GFP^+^CD33^+^ cells among total live leukocytes was calculated to quantify the level of leukemic cell infiltration in the peripheral blood.

### 4.11. Pathological and Histological Analysis

Liver and spleen tissues were harvested, weighed, and fixed in 4% paraformaldehyde. Following fixation, tissues were dehydrated and embedded in paraffin using an automatic tissue dehydrator (JT-12J, Wuhan Junjie Electronics, Wuhan, China)) and a paraffin embedding machine (JB-P5, Wuhan Junjie Electronics). Xylene (cat. no. 10023418, Sinopharm, Shanghai, China) was used as a clearing agent. Tissue blocks were sectioned at a 4 μm thickness using a pathological microtome (Leica RM 2016, Leica Biosystems, Nussloch, Germany). Sections were mounted onto adhesion slides (PC2-301-16, Meiweide Life Science, Guangzhou, China), dried at 60 °C for 3 h on a tissue drying plate (JK-6, Wuhan Junjie Electronics), then deparaffinized in xylene and rehydrated through a graded ethanol series. Staining was performed with Mayer’s hematoxylin (cat. no. H9627, Sigma) for 5 min, followed by 1% alcoholic eosin (cat. no. 71014460, Sinopharm) for 2 min. After dehydration and clearing, sections were mounted with neutral balsam (cat. no. 20200237, Nanchang Yulu Experimental Equipment, Nanchang, China). Images were captured using a light microscope (Nikon ECLIPSE Ci-L) equipped with a digital camera (Nikon Fi3, Nikon Corporation, Tokyo, Japan). Leukemic infiltration was assessed by light microscopy based on the morphological identification of NB4-like cells (large nuclei, high nuclear-to-cytoplasmic ratio, fine chromatin, and prominent nucleoli) in hematoxylin–eosin-stained liver and spleen sections. Two independent observers blinded to the groups performed the evaluation. Representative images were captured to illustrate the presence or absence of infiltrating blasts.

### 4.12. Analysis of APL Patient Cohorts

The clinical relevance of TCEA1 in APL was evaluated using the Kaplan–Meier Plotter online tool (https://kmplot.com/), which integrates gene expression and survival data from the Gene Expression Omnibus (GEO) and The Cancer Genome Atlas (TCGA) [29,30]. The analysis was restricted to patients with the FAB M3 subtype (APL). Patients were dichotomized into high- and low-expression groups using the platform’s optimal cutoff algorithm, which identifies the expression threshold that maximizes the survival difference. The optimal cutoff value was 3975. Patients with TCEA1 expression above 3975 were assigned to the high-expression group, and those below or equal to 3975 to the low-expression group. Kaplan–Meier survival curves were generated, and the log-rank test was used to compare overall survival between the two groups. The hazard ratio (HR) with the 95% confidence interval (CI) was calculated using the Cox proportional hazards regression model.

### 4.13. Statistical Analysis

Data are presented as mean ± SD. Western blot band intensities were quantified using ImageJ software and normalized to the loading control. Comparisons between the two groups were made using a two-tailed, unpaired Student’s *t*-test. Survival curves were analyzed by the log-rank test. *p* values < 0.05 were considered significant. Analyses were performed using GraphPad Prism 11.

## Data Availability

All data generated in this study are available from the author and the Kaplan–Meier Plotter online tool (https://kmplot.com/), which are publicly available resources.

## Supplementary Materials

The following supporting information can be downloaded at: https://www.mdpi.com/article/10.3390/ijms27125380/s1.

## Abbreviations

The following abbreviations are used in this manuscript:

AML	Acute Myeloid Leukemia
APL	Acute Promyelocytic Leukemia
C/EBPε	CCAAT/Enhancer-Binding Protein Epsilon
IRF8	Interferon Regulatory Factor 8
ELANE	Neutrophil Elastase
MMP9	Gelatinase
MPO	Myeloperoxidase
PRTN3	Proteinase 3
TCEA1	Transcription Elongation Factor A (SII) 1
LTF	Lactoferrin
CRISP3	Cysteine-Rich Secretory Protein 3
RNAPII	RNA Polymerase II
PML-RARα	Promyelocytic Leukemia/Retinoic Acid Receptor Alpha
ATRA	All-Trans Retinoic Acid
ATO	Arsenic Trioxide
G-CSF	Granulocyte Colony-Stimulating Factor
IL-3	IL-3
32Dcl3	Murine Myeloid Progenitor Cell Line
HE	Hematoxylin and Eosin
