# Supplementary material for: TCEA1 Suppresses Acute Promyelocytic Leukemia by Upregulating C/EBPε and IRF8
Source: Int J Mol Sci. 2026 Jun 15;27(12):5380. doi: 10.3390/ijms27125380 (PMC13300213; doi:10.3390/ijms27125380)
Supplement: Supplementary file 1 [file ijms-27-05380-s001.zip › ijms-4330565-supplementary.pdf]

Supplement Table S1. PCR and qPCR primers sequence.

Primer No	Primer name	Primer sequence
1	C/EBPε-F	AAGCTTGGTACCGAGCTCGGATCCGCCACCATGTCCCACGGGACCT
	C/EBPε-R	GCGGGTTTAAACGGGCCCTCTAGATCAGCTGCAGCCCCCG
2	IRF8-F	AAGCTTGGTACCGAGCTCGGATCCGCCACCATGTGTGACCGGAACG
	IRF8-R	GCGGGTTTAAACGGGCCCTCTAGATTAGACGGTGATCTGTTGATTT
3	TCEA1-F	CTAGAGGATCTATTTCCGGTGAATTCGCCACCATGGAGGACGAAGTGGTCCG
	TCEA1-R	GCAGATCCTTACTAGTATCGATGGATCCTCAACAGAACTTCCATCGAT
4	GAPDH-F	GAGAGTGTTTCCTCGTCCCGTA
	GAPDH-R	CCTCACCCCATTTGATGTTAGT
5	C/EBPε-F	CCAGTCGAGGCAGCTACAAT
	C/EBPε-R	ATAATGCGCCTCTTGGCCTT
6	IRF8-F	GCAGGTCTTTGACACCAACCA
	IRF8-R	AAGGGTCTCTGGTGTGAGGTA
7	Rps16-F	CCCCTGGAGATGATTGAGCC
	Rps16-R	CACCACCCTTTACACGGACA
8	MPO-F	CGCCCAACAACATCGACATC
	MPO-R	ATGCTGAACACACCCTCGTT
9	PRTN3-F	CGCGGAGAACAAACTGAACG
	PRTN3-R	CCATCACAGATCAGGGGGC
10	ELANE-F	TCTTTTCCTCGCCTGTGTCC
	ELANE-R	GGACGTTTACATTCGCCACG
11	LTF-F	GTCTTCCTCGTCCTGCTGTT
	LTF-R	CATTTTGTGGCCTCGGGTTG
12	MMP9-F	TCTATGGTCCTCGCCCTGAA
	MMP9-R	CATCGTCCACCGGACTCAAA
13	CRISP3-F	TCTGGAAACCACTGCAATGAC
	CRISP3-R	TTTGGGTTACTGTGTCTGTA
